# Assessing the potential of composite confining systems for secure and long-term CO_2_ retention in geosequestration

**DOI:** 10.1038/s41598-023-47481-2

**Published:** 2023-11-29

**Authors:** Sahar Bakhshian, Alexander P. Bump, Shaunak Pandey, Hailun Ni, Susan D. Hovorka

**Affiliations:** Bureau of Economic Geology, Jackson School of Geosciences, The University of Texas at Austin, Austin, TX USA

**Keywords:** Carbon capture and storage, Computational science

## Abstract

A potential geologic target for CO_2_ storage should ensure secure containment of injected CO_2_. Traditionally, this objective has been achieved by targeting reservoirs with overlying seals-regionally extensive, low permeability units that have been proven capable of retaining buoyant fluid accumulations over geologic time. However, considering that the amount of CO_2_ is limited by a decadal injection period, vertical migration of CO_2_ can be effectively halted by a composite system of discontinuous shale/silt/mudstone barriers in bedded sedimentary rocks. Here, we studied the impact of depositional architectures in a composite confining system on CO_2_ migration and confinement at reservoir scale. We stochastically generated lithologically heterogeneous reservoir models containing discontinuous barriers consistent with statistical distributions of net-sand-to-gross-shale ratio (*NTG*) and horizontal correlation lengths derived from well log data and observations of producing hydrocarbon fields in Southern Louisiana. We then performed an extensive suite of reservoir simulations of CO_2_ injection and post-injection to evaluate the sensitivity of CO_2_ plume migration and pressure response of the composite system to a series of geologic and fluid parameters including the lateral continuity of barriers, *NTG*, permeability anisotropy within the sand body, and capillary pressure contrast between the sand and shale facies. The results indicate that lateral continuity of barriers and *NTG* are the dominant controls on CO_2_ plume geometry and pressure build-up in the reservoir, while the impact of *NTG* is particularly pronounced. The significance of intraformational barriers becomes apparent as they facilitate the local capillary trapping of CO_2_. Those barriers improve the pore space occupancy by promoting a more dispersed shape of the plume and ultimately retard the buoyancy-driven upward migration of the plume post injection.

## Introduction

CO_2_ storage capacity can be highly controlled by the heterogeneity of the storage formations, encompassing both structural (e.g., fractures and faults) and stratigraphic (e.g., sand-body continuity, permeability, and porosity) heterogeneity. Heterogeneity of geologic formations may manifest itself with different characteristics across different scales. At the reservoir scale, stratigraphic heterogeneity appears as distinct lithofacies, creating intraformational barriers in sand bodies^1^. Intraformational barriers primarily comprise fine-grained facies such as mudstones, siltstones, and shales with low porosity and permeability and medium to high capillary entry pressure. Thus, those barriers can impact reservoir connectivity and control the migration pathway of fluids^2–4^. This multi-layer system of discontinuous stratigraphic barriers has been defined as a composite confining system by Bump et al.^5^. In the context of CO_2_ storage applications, flow barriers can act as impediments to the buoyancy-driven upward migration of CO_2_ plume, redirecting its migration primarily in a lateral direction^6–11^.

To have a successful CO_2_ storage project, the potential geologic target should be selected such that it secures sufficient containment of CO_2_ plume^12^. Traditionally, regional seals called caprocks with high capillary entry pressure capable of holding a large column of CO_2_ are considered key elements for containment of CO_2_^13,14^. However, a large volume of CO_2_ can be locally trapped beneath the localized intraformational barriers composed of fine-grained facies, without relying on the continuity of individual beds. The invasion of injected CO_2_ through these barriers is impeded by the capillary pressure contrast between the sandbody and fine-grained facies^8,15^. Existing reservoir modelings, experimental studies, and field-scale observations distinctly confirm the effectiveness of capillary trapping in the confinement of CO_2_ plume^5,6,16–19^. The trapping mechanism of CO_2_ beneath the flow barriers is called capillary heterogeneity trapping induced by the capillary pressure contrast between the sandbody and fine-grained barriers^20,21^. Local trapping of CO_2_ under the intraformational barriers is equivalent to the capillary pinning mechanism^22,23^ that retards the buoyancy-driven migration of CO_2_ plume to shallow depths of the reservoir and with a good design, the composite confining system will prevent CO_2_ from reaching the top of the confining system. In addition, the tortuous flow path of CO_2_ plume created by the existence of discontinuous barriers causes a large volume of rock and resident brine to be in contact with CO_2_, and hence, facilitates other CO_2_ trapping mechanisms such as snap-off, dissolution, and mineral trapping. Capillary heterogeneity trapping of CO_2_ controlled by intraformational barriers has been demonstrated as an effective mechanism for CO_2_ containment in previous studies^15,17,18,24^.

The heterogeneity in the spatial distribution and lateral continuity of discontinuous shale barriers as well as sand/shale ratios are among the major uncertainty associated with the composite confining systems^5,25^. These stratigraphic heterogeneities cannot be resolved deterministically using seismic data. Additionally, quantifying the lateral heterogeneity of shale barriers using well-to-well correlation data is challenging as the data are sparse due to the large spacing between the wells. Thus, correlating the stratigraphic distribution of barriers between the wells using deterministic modeling is difficult and interpretations are not typically distinctive^25^. Efforts have been undertaken to utilize stochastic techniques in lieu of conventional mapping to model complex heterogeneity distribution within a heterogeneous reservoir^25,26^. The main intention of this study is to consider a wide range of lateral continuity and density of shale barriers in geologic models and investigate the impact of these architectures and their characteristics on the migration and confinement of CO_2_ within these formations. To this end, we employed a stochastic modeling approach to generate heterogeneous facies models containing horizontal discontinuous shale barriers populated in sand bodies, controlled by the correlation length of barriers and sand/shale ratios as factors constrained by Spontaneous Potential (SP) well-log data and observations of producing hydrocarbon fields^5^.

In this study, we performed a set of reservoir simulations involving CO_2_ injection and post-injection in a composite confining system to evaluate the sensitivity of CO_2_ plume migration and pressure response of the system to a range of geologic and fluid parameters. Net-sand-to-gross-shale ratio (*NTG*) and horizontal correlation lengths of shale barriers were considered as factors controlling the degree of heterogeneity in the geologic models. The composite confining models generated in this study serve as representative examples of Southern Louisiana Miocene deltaic deposits with large numbers of interbedded barriers and no regionally extensive caprock seals. To simplify the model, we did not account for a distinct injection zone commonly considered in actual storage projects. Instead, we injected CO_2_ into the lower section of the confining zone. Using the reservoir simulation results, the performance of a composite system for CO_2_ confinement has been evaluated and the corresponding sensitivity analysis results have been presented in the following sections.

## Methods

### Geostatistical model

We used a synthetic geologic model to perform a sensitivity analysis for characterizing CO_2_ migration and confinement in a composite confining system. To mimic a composite system of flow barriers, we have geostatistically generated two-dimensional (2D) heterogeneous facies models containing horizontal discontinuous shale barriers (i.e., capillary barriers) populated in sand bodies. The models involve 150 × 150 grid blocks with horizontal and vertical resolutions of 219 ft and 2.2 ft, respectively. The shale barriers were considered to be laterally correlated with correlation lengths ranging from 1973 ft (600 m, equivalent to 6% of the model’s horizontal extent) to 26,212 ft (8 km, equivalent to 80% of the model’s horizontal extent). The stochastic facies models were constrained by two metrics including statistical distribution of net-to-gross ratio (*NTG*) and horizontal correlation length of shale barriers. The distribution of *NTG* ratio was derived from evaluation of Spontaneous Potential (SP) logs from 323 wells in Southern Louisiana that record over 40,000 individual mudstones within the Miocene deltaic and shore zone facies. Log values were normalized to a range of −80 to +20 MV and a cutoff value of − 30 MV was employed to distinguish between sandy and muddy facies. The footprint of the hydrocarbon-water contacts was used to estimate the range of barrier lengths^5^.

The spatial distribution of shale facies within the 2D models is generated using a spectral method^27^. First, we generated a white random noise matrix of $\mathfrak {R(\overrightarrow{r})}$ with the size of 150 × 150. Then, we calculated its Fourier transform $\mathscr {F(\overrightarrow{r})}$, multiplied by a Gaussian function:

1 $$\begin{aligned} \mathscr {G(\overrightarrow{s})} = \alpha \mathscr {F(\overrightarrow{s})}e^{-\frac{|\textbf{s}|^2}{s_0^2}}, \end{aligned}$$

By taking the inverse Fourier transform of $\mathscr {G(\overrightarrow{s})}$, we obtained a correlated Gaussian distribution field, $\mathscr {R(\overrightarrow{r})}$, yielding an auto-correlation function:

2 $$\begin{aligned} (\mathscr {R} ^* \mathscr {R})(x) \propto e^{-\frac{s_0^{2}}{8} x^2} = e^{-\frac{1}{2} (\frac{\pi x}{2 \lambda })^2}, \end{aligned}$$

Where $*$ denotes the convolution product and $\lambda = {\pi }/{s_0}$ represents the correlation length considered for the shale facies ($cl_x$). For a given shale/sand ratio (*NTG*), the grid blocks corresponding to shale facies are defined as $\mathscr {R(\overrightarrow{r})}_s = \left\{ \overrightarrow{r}\mid \mathscr {R(\overrightarrow{r})} > NTG\right\}$.

**Figure 1:**
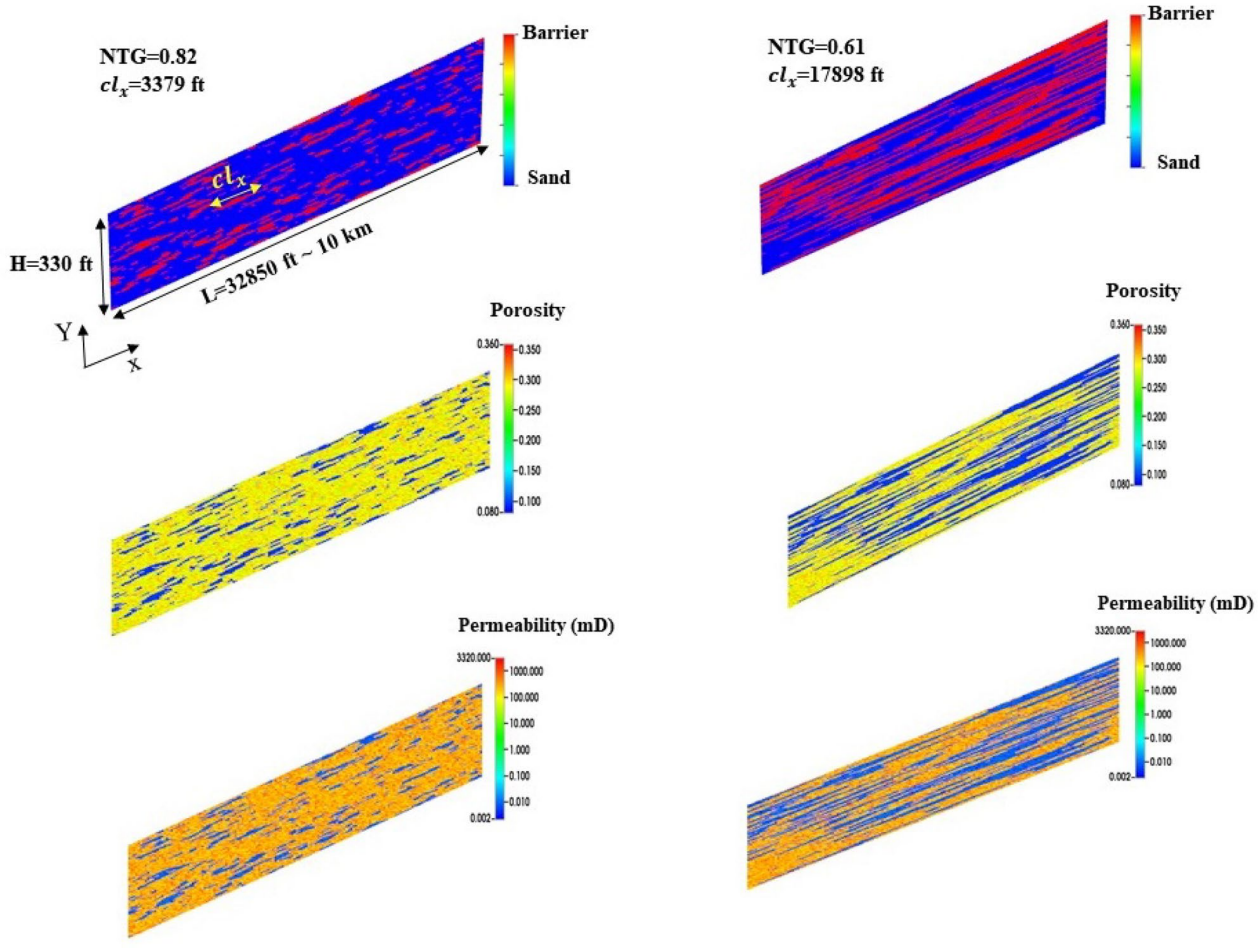
Two examples of geostatistical models with different net-to-gross (*NTG*) ratios and correlation lengths of impermeable barriers in the horizontal direction ($cl_x$). The model extends 330 ft and 32,850 ft in the vertical and horizontal directions, respectively. The upper figures represent the facies distributions. Blue is sand and red represents impermeable barriers. The middle figures represent the porosity distributions. The lower figures show permeability distributions.

**Figure 2:**
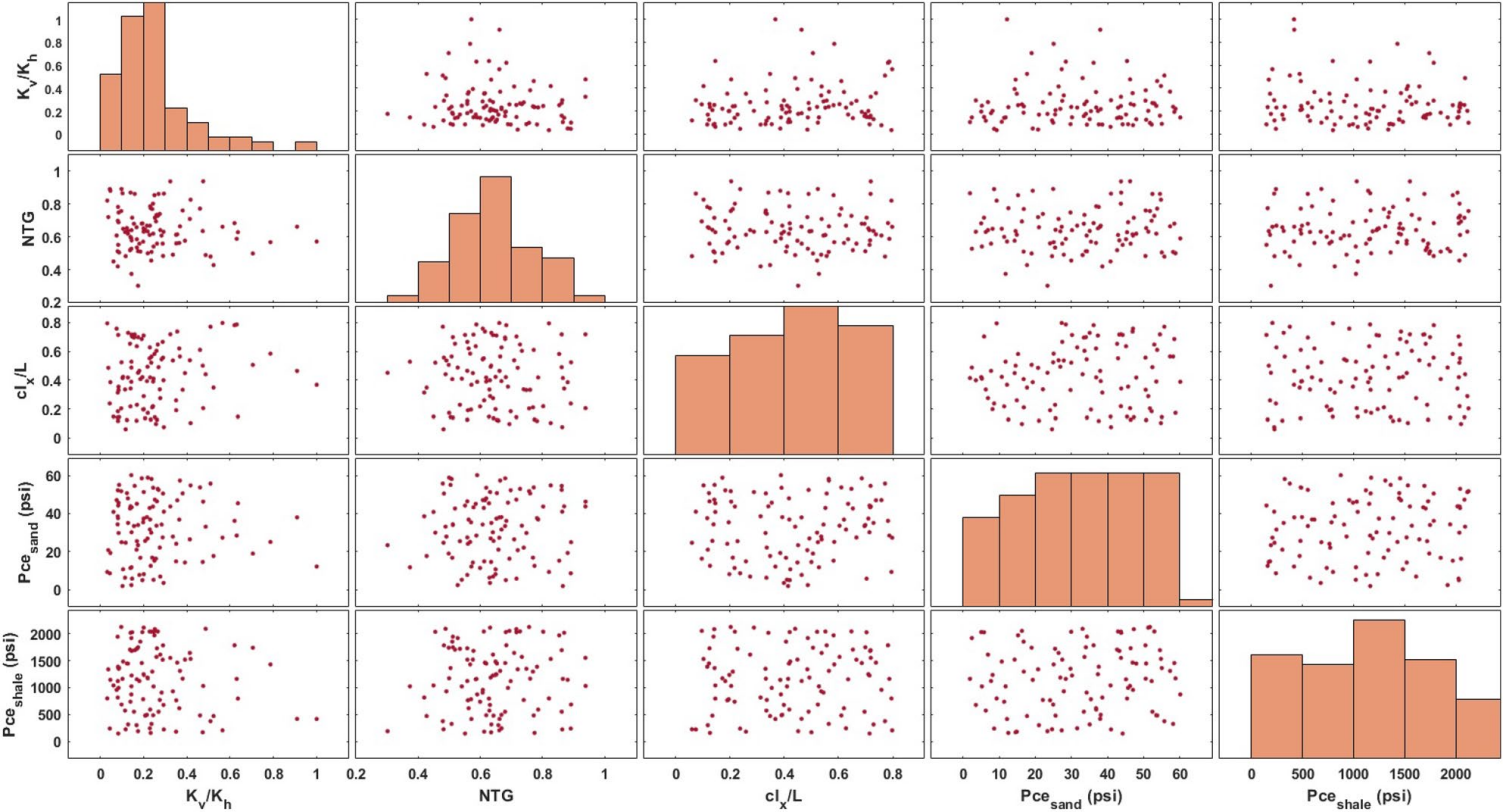
Paired scatter plot illustrating the distribution of five uncertain parameters including *NTG*, $cl_x$, $K_{v}/K_{h}$, $P_{ce,sand}$, and $P_{ce,shale}$, used for the sensitivity analysis. The x-axis and y-axis of each plot represent two different variables of interest. 100 different combinations of these uncertain parameters were sampled from their distributions to create various stochastic reservoir models.

**Figure 3:**
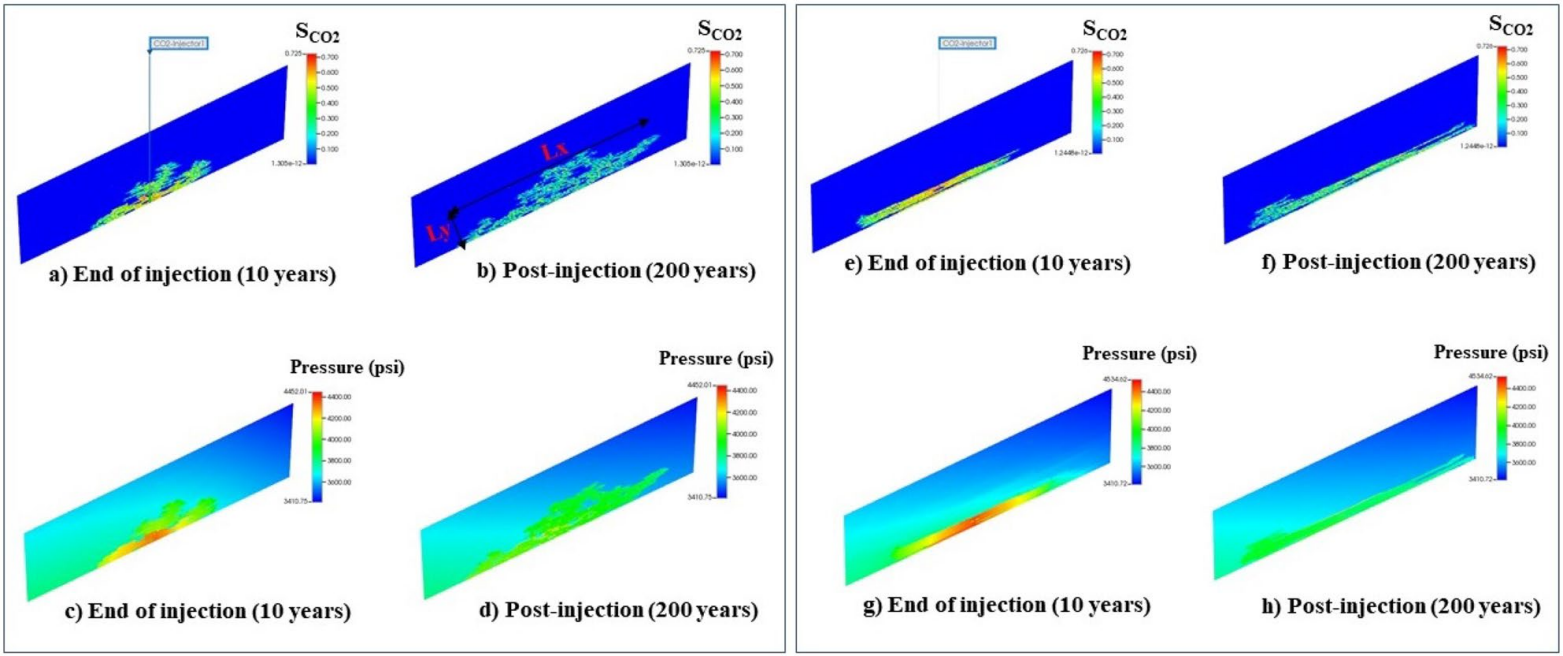
$S_{CO_2}$ saturation and pressure profile at the end of injection and 200 years of post-injection for two geologic models shown in Fig. 1. The figures in the left box (**a**–**d**) correspond to *NTG* = 0.82 and $cl_x$ = 3379 ft and the figures in the right box (**e**–**h**) correspond to *NTG* = 0.61 and $cl_x$ = 17,898 ft. $L_x$ and $L_y$ are the maximum extension of $S_{CO_2}$ plume in the horizontal and vertical directions, respectively.

The horizontal permeability field for each facies was generated using a log-normal distribution using the mean values of 500 mD and 0.01 mD for sand and shale, respectively. The standard deviation of the natural log transform of permeability was assumed to be 0.45 for both facies. The permeability field of sand was considered anisotropic by taking the vertical-to-horizontal permeability ratio ($K_v/K_h$, where $K_v$ and $K_h$ are the vertical and horizontal permeability, respectively) as one of the uncertain parameters in this study. The porosity ($\phi$) distribution is assumed to be dependent on permeability as follows^28^:

3 $$\begin{aligned} K_h=7\times 10^7 (\phi ^{9.61})\;, \end{aligned}$$

The spatial distribution of porosity and permeability in two instances of stochastic geologic models with different *NTG* and $cl_x$ is displayed in Fig. 1.

### Reservoir model

We used an Equation-of-State (EoS) reservoir simulator for compositional modeling, namely, $\hbox {GEM}^{\textrm{TM}}$ [Computer Modelling Group Ltd. (CMG), Calgary, Alberta, Canada]^29^ to simulate CO_2_ storage in saline aquifers. Two-dimensional reservoir models with dimensions of 32,850 ft and 330 ft in the horizontal and vertical directions are generated using a stochastic method. The reservoir’s lateral dimension is taken to be sufficiently large for better monitoring of the CO_2_ plume lateral extension in a long-term simulation. The upper and lower boundaries of the reservoir were considered closed, while open boundaries are imposed on the lateral sides of the model by extending the pore volume of the outer boundary grid blocks. The model has a dip of 1$^{\circ }$ and depth of 8000 ft. The initial pressure and temperature of the reservoir were assigned using a hydrostatic gradient of 0.465 psi/ft and a geothermal gradient of 0.0165 F/ft, with surface pressure and temperature set at 14.7 psi and 60 F, respectively^30^.

The Brooks-Corey model was used to generate the relative permeability curves of water and CO_2_ ($k_{rw}$, $k_{rg}$), given by^31,32^

4 $$\begin{aligned} k_{rw}&=({S_{w}^*})^{4}\;, \end{aligned}$$

5 $$\begin{aligned} k_{rg}&=0.4[1-(\hat{S_{w}})^2](1-\hat{S_{w}})^2 \;, \end{aligned}$$

6 $$\begin{aligned} S_{w}^*&=\frac{S_w - S_{wi}}{1 - S_{wi}}\;, \end{aligned}$$

7 $$\begin{aligned} \hat{S_{w}}&=\frac{S_w - S_{wi}}{1 - S_{wi}-S_{gr}}\;, \end{aligned}$$

Where $S_{w}$ denotes the water saturation. The irreducible water saturation ($S_{wi}$) and the critical CO_2_ saturation ($S_{gr}$) are taken to be 0.2 and 0.05, respectively. To account for CO_2_ residual trapping, we incorporated hysteresis characteristics into the relative permeability curve. To model the gas relative permeability hysteresis, we applied the Land trapping model, which relates the trapped gas saturation $S_{gt}$ to the initial gas saturation $S_{gi}$ during the imbibition as^33^

8 $$\begin{aligned} S_{gt}=\frac{S_{gi}}{1+C S_{gi}} \end{aligned}$$

Where *C* is the Land’s trapping coefficient calculated as follows:

9 $$\begin{aligned} C=\frac{1}{S^{max}_{gr}}-\frac{1}{S^{max}_{g}} \end{aligned}$$

Where $S^{max}_{g}$ is the maximum gas saturation associated with the imbibition curve, and $S^{max}_{gr}$ is the maximum residual gas saturation. In this study, the $S^{max}_{gr}$ is assumed to be 0.2, and $S^{max}_{g}=1-S_{wi}$. We did not quantify the uncertainty surrounding the impact of relative permeability on CO_2_ plume geometry and reservoir pressure. Nevertheless, previous studies have demonstrated the effect of relative permeability parameters on pressure accumulation and CO_2_ plume configuration in homogeneous reservoirs^34,35^.

The drainage capillary pressure was created using the Brooks-Corey model as follows:

10 $$\begin{aligned} P_c=P_{ce} \times \left(\frac{S_{w}-S_{wi}}{1-S_{wi}} \right)^{-0.5}\;, \end{aligned}$$

Where $P_{ce}$ is the capillary entry pressure. $P_{ce}$ is considered as one of the uncertain parameters, ranging from 0.03 psi to 60.2 psi for sand ($P_{ce,sand}$) and 137 psi to 2146 psi for shale ($P_{ce,shale}$)^36^. We did not account for hysteresis in the capillary pressure curves.

To consider the effect of local heterogeneity on capillary pressure, we employed the Leverett *J*-function^37^

11 $$\begin{aligned} J (S_{w})=\frac{P_c(S_{w})\sqrt{K_h/\phi }}{\sigma cos\theta }\;, \end{aligned}$$

Where $\sigma$ is interfacial tension and $\theta$ is contact angle. Using the *J*-function, we scaled the capillary pressure curve for each grid block using their porosity and permeability values, while $\sigma$ and $\theta$ are considered spatially invariant.

### Design of experiments

We conducted CO_2_ injection simulations in geologic models by considering 100 different combinations of uncertain parameters, namely, *NTG*, $cl_x$, $K_{v}/K_{h}$, $P_{ce,sand}$, and $P_{ce,shale}$. The range of variations for each uncertain parameter is shown in Fig. 2. For all simulations, supercritical CO_2_ was injected through a single well for a period of 10 years with an injection rate of 0.7 mmscf/day and a maximum well’s bottom-hole pressure of 6400 psi. The maximum allowable injection pressure was selected to be 80$\%$ of the rock fracture pressure, which is estimated to be 8000 psi based on the lithostatic pressure gradient of 1 psi/ft. The injection rate and period were chosen to manage maximum pressure build-up and maintain stabilized injectivity. The injection well is located in the center of the domain with a perforation length of 44 ft equivalent to grid blocks of 130 to 149 in the vertical direction. To simplify the model, we did not account for a distinct injection zone commonly considered in actual storage projects. Instead, we injected CO_2_ into the lower section of the confining zone. The simulations continued for 200 years after injection stopped. One criterion for selection of 200-year post-injection simulations was to ensure the stabilization of CO$_2$ upward migration.

We quantified the sensitivity of CO$_2$ plume migration and pressure response of the reservoir to fluid/geologic input features. The vertical movement of the CO$_2$ plume is quantified with the height of the center of mass of the plume (in the *y* direction) by calculating the first spatial moment, defined as^38^

12 $$\begin{aligned} M_{i,j}&=\int _{0}^{L} \int _{0}^{H} \phi (x,y) \rho (x,y)s(x,y) x^iy^j\,dx\,dy \; \end{aligned}$$

13 $$\begin{aligned} CM&=\frac{M_{01}}{M_{00}} \; \end{aligned}$$

Where $\rho$ is the CO$_2$ density and *s* denotes CO$_2$ saturation in each grid block. *x* and *y* are the coordinates of grid blocks and *L* and *H* are respectively the lateral extent and thickness of the geologic model.

The response metrics were selected as the average grid-based saturation of CO$_2$ plume ($S_{CO_2}$), the maximum dimensionless lateral ($L_x/L$) and vertical ($L_y/H$) extent of the CO$_2$ plume, where $L_x$ and $L_y$ are the maximum extent of the plume in the *x* and *y* directions, respectively, dimensionless center of mass of the CO$_2$ plume (*CM*/*H*), maximum pressure buildup in the reservoir ($\Delta P_{max}$), the pressure buildup at the reservoir top ($\Delta P_{top}$), and the dimensionless depth at which the maximum pressure buildup occurs ($H_{max}/H$). The response variables were measured at the end of injection and 200-year post-injection. Our intention was to capture the viscous- and buoyancy-driven migration of CO$_2$ occurring during injection and post-injection, respectively. Thus, the simulations were conducted for 10 years of injection and continued for 200 years after injection stopped.

## Results and discussion

### CO$_2$ saturation and pressure plume dynamics

**Figure 4:**
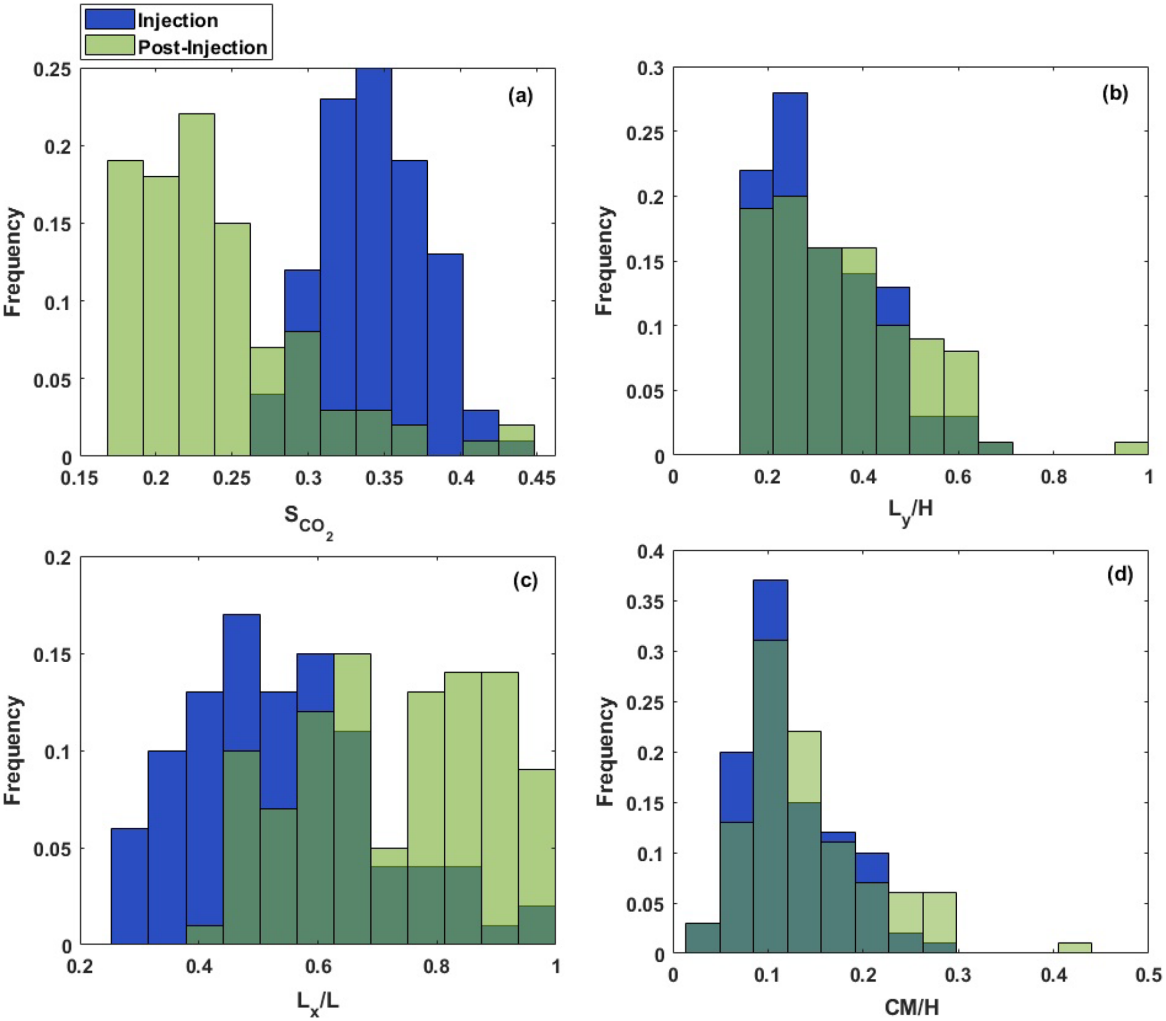
Probability distribution of output variables including the average CO$_2$ saturation ($S_{CO_2}$), dimensionless height of the plume ($L_y/H$), dimensionless lateral extension of the plume ($L_x/L$), and center of mass of the plume (*CM*/*H*) at the end of injection and 200 years post-injection.

**Figure 5:**
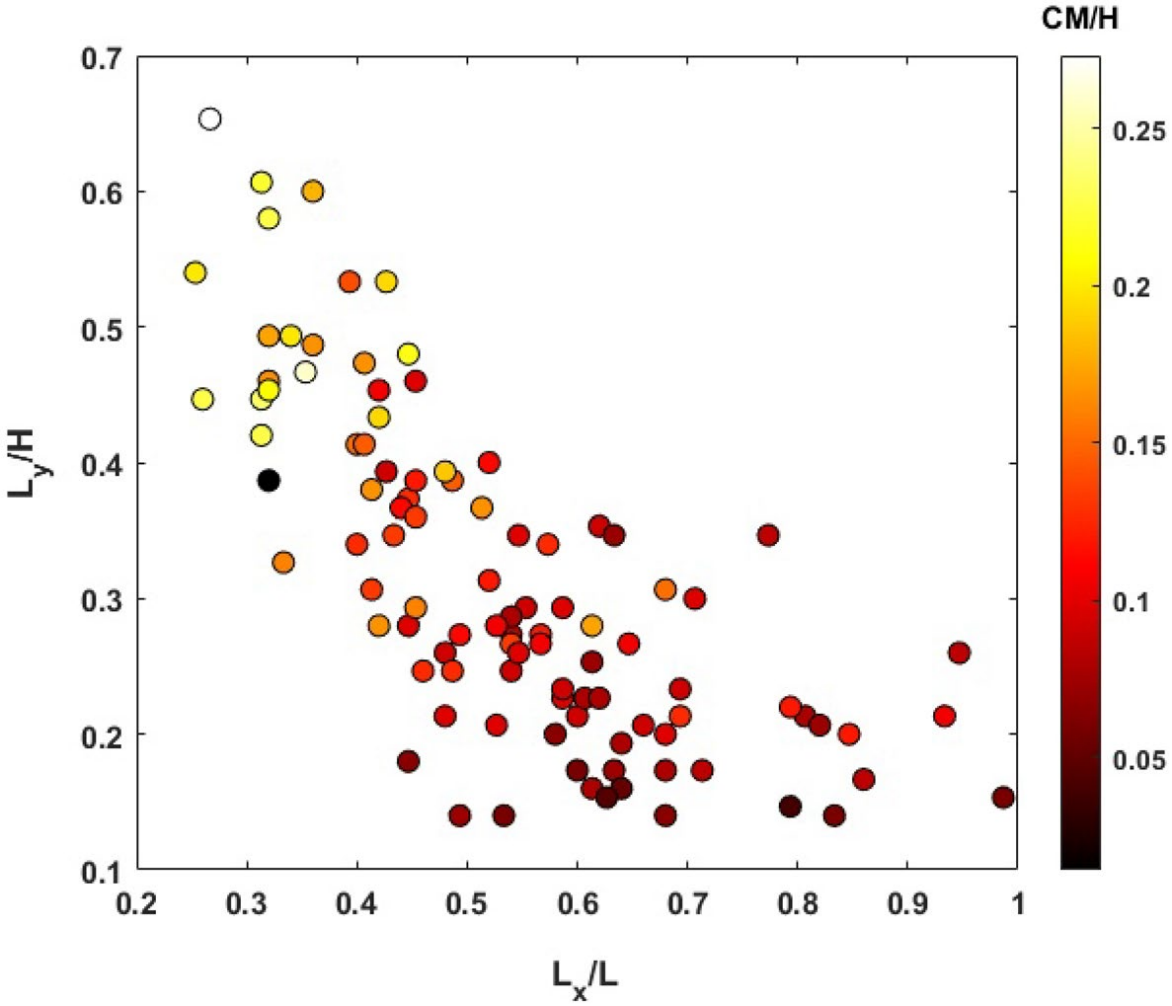
Correlation between the plume height ($L_y/H$) and its lateral extension ($L_x/L$) and their relation to the plume center of mass (*CM*/*H*).

**Figure 6:**
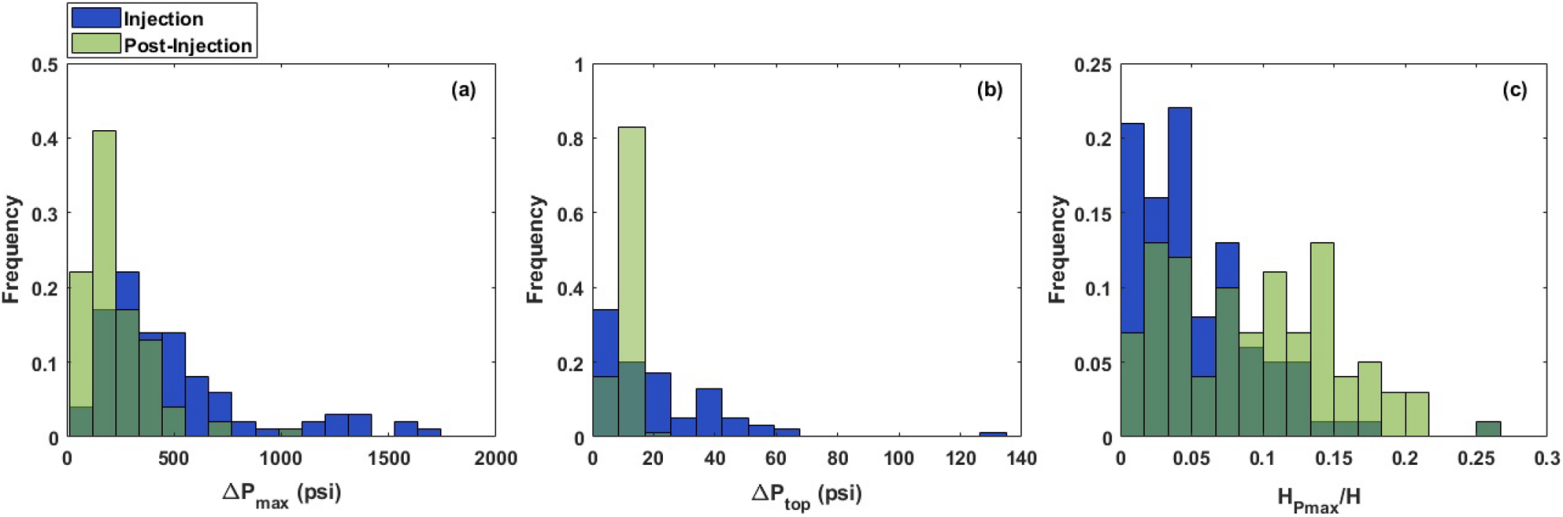
Probability distribution of the pressure-related output variables including the maximum pressure buildup ($\Delta P_{max}$), the pressure buildup at the top of the composite system ($\Delta P_{top}$), and the location of the pressure buildup occurrence ($H_{pmax}/H$) at the end of injection and 200 years post-injection.

We investigated the migration of CO$_2$ plume and pressure response of the confining system under various scenarios considering different combinations of uncertain variables. Through this parametric sensitivity analysis, we were able to determine the combined role of uncertain variables on CO$_2$ saturation and pressure plume behavior. Figure 3 displays CO$_2$ saturation and pressure profile at the end of injection and 200 years of post-injection for two geologic models with various *NTG* and $cl_x$ values, representing how heterogeneity can affect the shape of the plume and distribution of pressure buildup in the reservoir. Figure 4 represents the probability distribution of response variables including $S_{CO_2}$, $L_y/H$, $L_x/L$ and *CM*/*H* obtained from injection and post-injection simulations using 100 combinations of uncertain parameters. The average CO$_2$ saturation of the plume was found to be 0.34 and 0.24 at the end of the injection and post-injection (200 years) stage, respectively. According to Fig. 4a, the distribution of $S_{CO_2}$ corresponding to the post-injection has shifted towards lower values and has a long skew towards higher values, indicating the spread or redistribution of CO$_2$ plume and occupying a larger pore volume of the pores. As shown in Fig. 3, CO$_2$ plume tends to become channelized underneath the capillary barriers and locally trapped with a saturation higher than the CO$_2$ residual saturation. These large saturation regions of CO$_2$ structurally trapped underneath the barriers by capillary heterogeneity potentially contain mobile CO$_2$^17^. This type of trapping is as effective as structural trapping unless the capillary integrity of those barriers is compromised through a large aperture feature such as an open wellbore.

Figure 4b and c display the probability of the lateral and vertical migration of CO$_2$ plume at the end of injection and late post-injection. According to those graphs, we observed that CO$_2$ plume propagates both laterally and vertically during the post-injection stage. However, the variation in its lateral expansion is more pronounced than in the vertical direction. This means that while the buoyancy effects promote the vertical migration of CO$_2$ plume at post-injection, the existence of barriers exerts a significant constraint on the influence of buoyancy forces and hence the vertical growth of the plume. In addition, according to Fig. 4b, there were a few case scenarios, in which the CO$_2$ plume reached the top of the formation ($L_y/H$ = 1). Increasing the thickness of the confining zone would likely increase the confinement capacity of those failed scenarios. In most scenarios, the plume mainly accumulated in the deeper parts of the confining zone, as the distributions have a long tail towards higher $L_{y}/H$ values. As shown in Fig. 4d, the location of the center of mass of the plume (*CM*) for all realizations did not exceed 50$\%$ of the reservoir thickness, meaning that CO$_2$ saturation tends to decrease towards the upper layers of the formation and the majority of the plume mass is concentrated in the deeper parts. Thus, the barriers facilitate the local trapping of CO$_2$ and retards its buoyancy-driven upward migration at the late post-injection. We also explored the correlation between the plume height and its lateral extension and showed how the center of mass of the plume varies with these two parameters (see Fig. 5). A negative correlation between the plume height ($L_{y}/H$) and its lateral extent ($L_{x}/L$) and positive correlation between $L_{y}/H$ and *CM*/*H* were observed.

**Figure 7:**
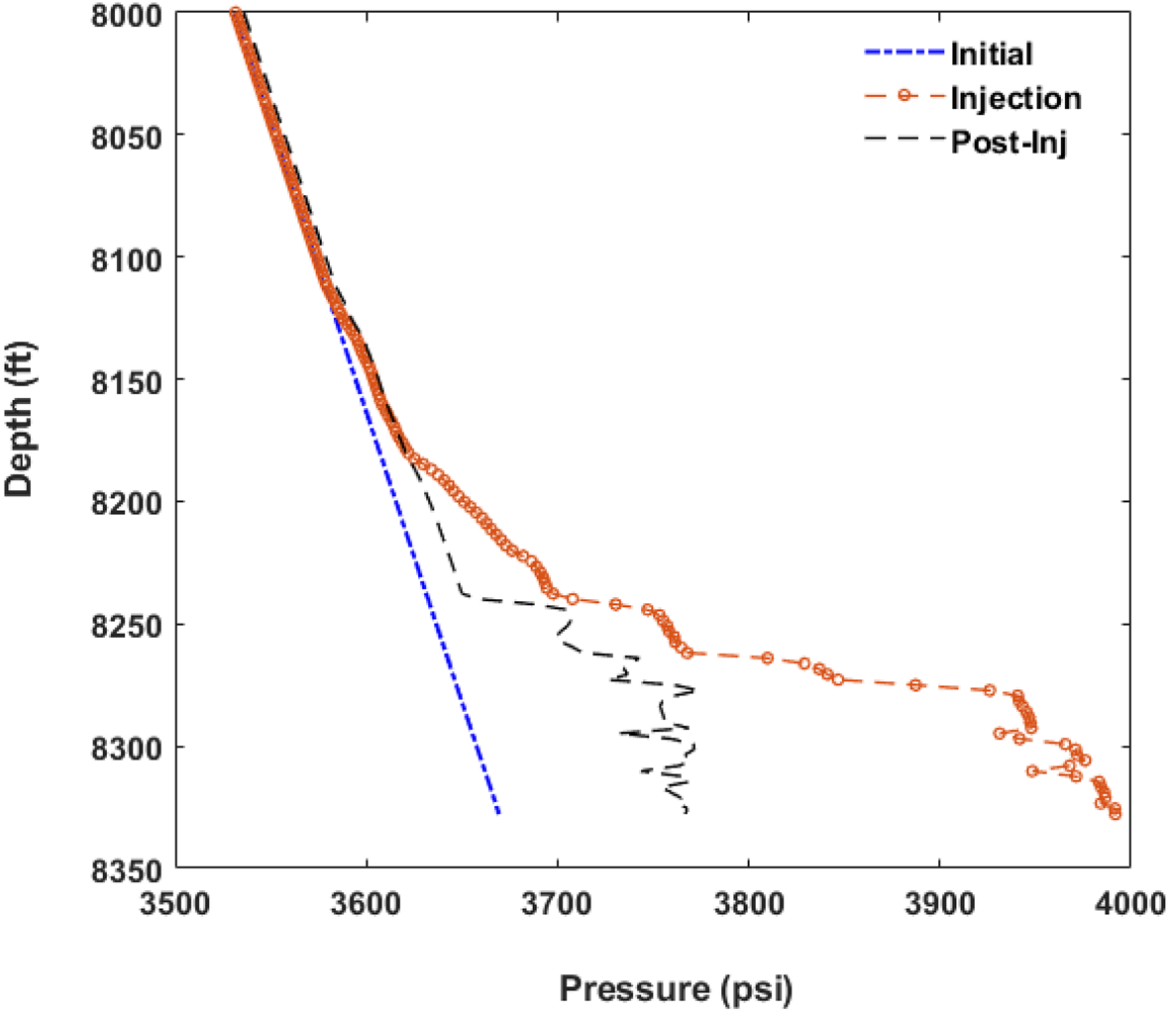
Pressure profile along the depth of the composite system in one of the case scenarios at the end of injection and 200 years post-injection.

**Figure 8:**
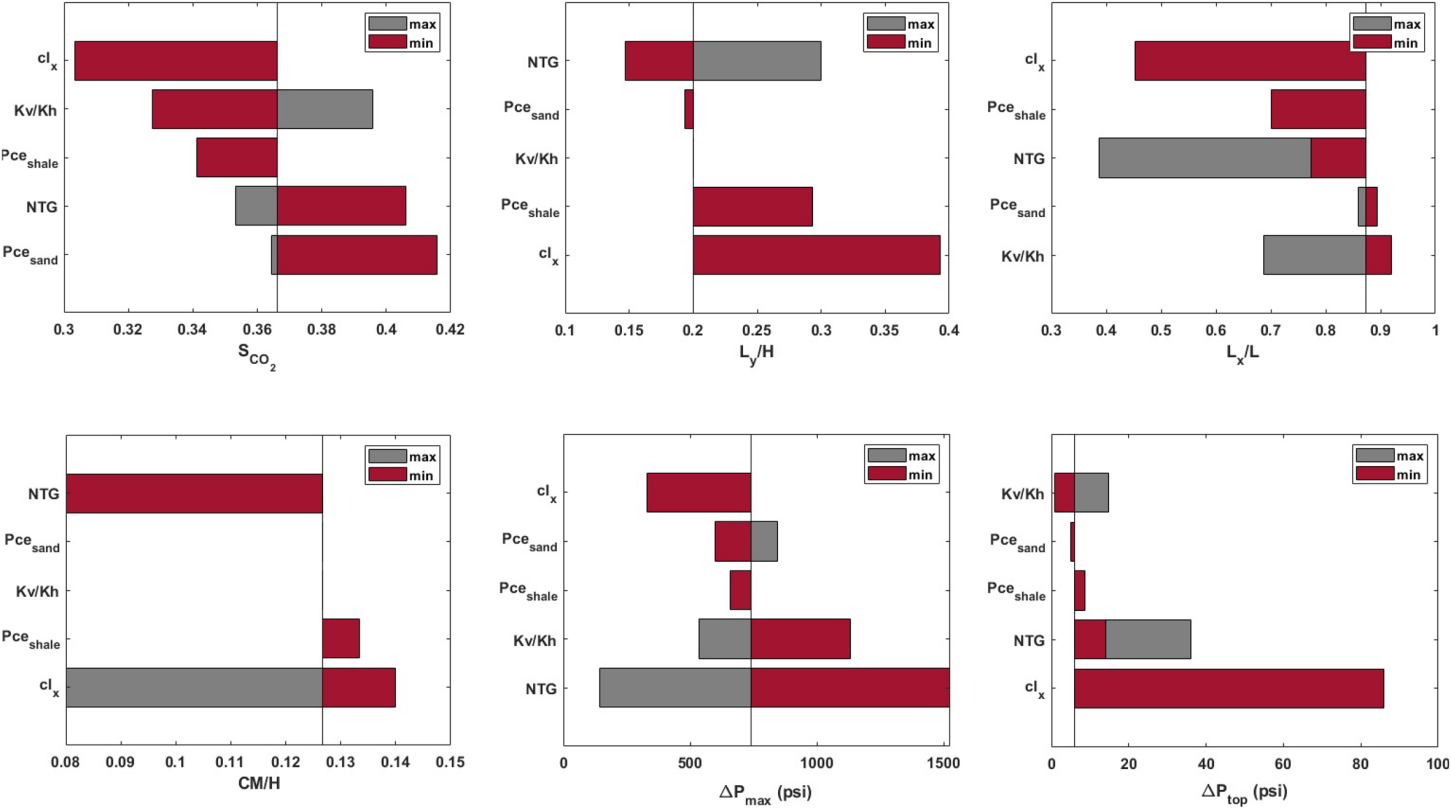
Tornado charts displaying the contribution of uncertain input parameters to the uncertainty of output parameters (model response at the end of injection).

**Figure 9:**
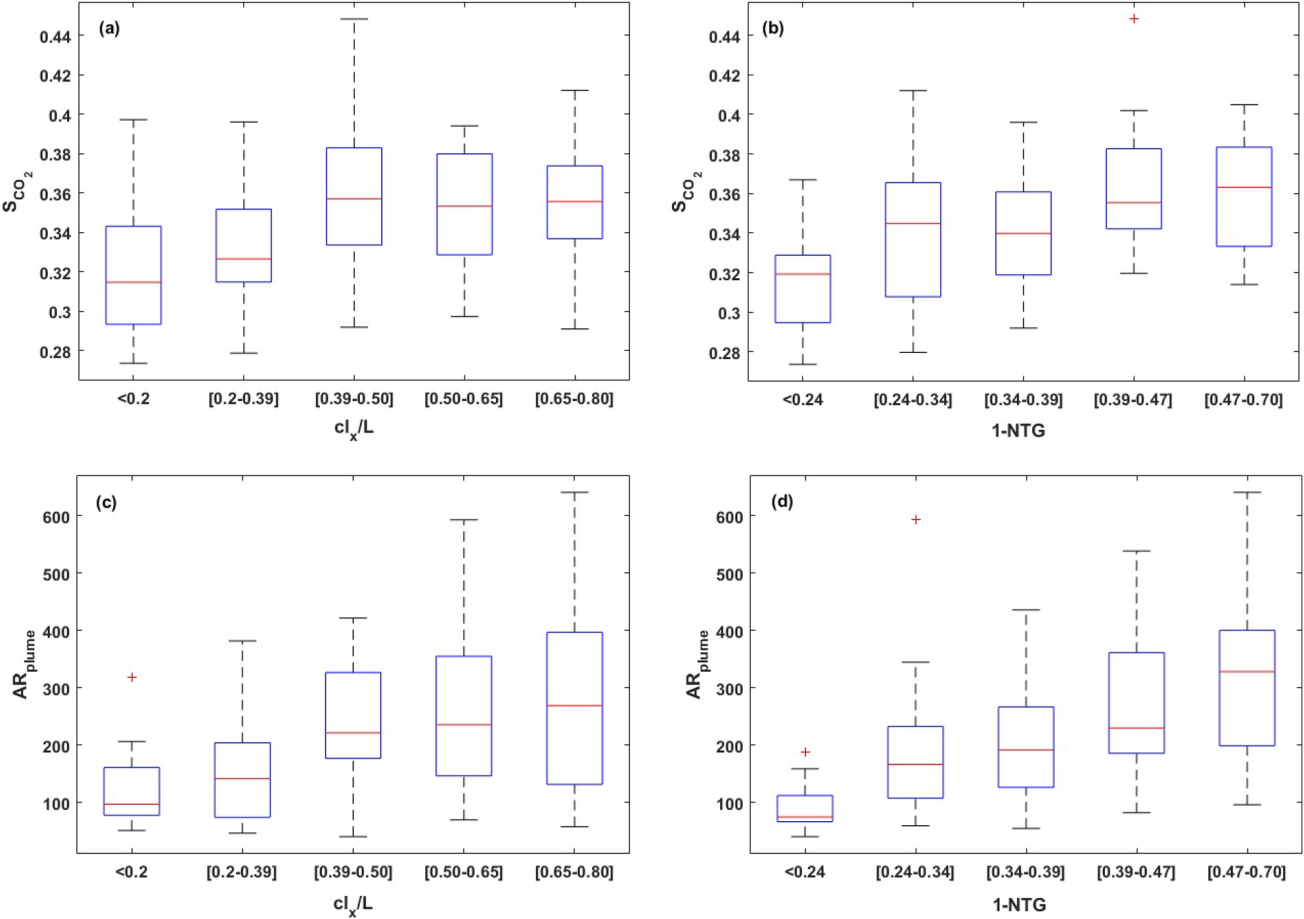
Box plots representing the dependence of CO$_2$ saturation ($S_{CO_2}$) and aspect ratio of the plume ($AR_{plume}$: which is $\frac{L_{x}}{L_{y}}$) on *NTG* and $cl_x/L$.

In addition to the analysis of the CO$_2$ saturation plume (i.e., the plume configuration), we further investigated pressure propagation in the reservoir at the end of injection and 200 years post-injection. Figure 6 represents the probability distribution of maximum pressure buildup ($\Delta P_{max}$), the pressure buildup at the top of the reservoir ($\Delta P_{top}$), and the location of the pressure buildup occurrence ($H_{pmax}/H$, where $H_{pmax}$ is the distance of the location of the maximum pressure buildup occurrence from the bottom of the reservoir) obtained from the injection and post-injection simulations using 100 realizations of uncertain parameters. As can be seen in Fig. 6a, $\Delta P_{max}$ distributions show the same trend for both end of injection and post-injection, as they both skew toward larger values. The average pressure buildup at the end of injection and post-injection was 500 psi and 228 psi, respectively. Additionally, according to the distributions shown in Fig. 6a, pressure attenuates significantly at the late post-injection, manifesting approaching an equilibrium state for the reservoir’s pressure after injection stops. As shown in Fig. 6b, the pressure buildup at the top of the reservoir is notably smaller than the maximum pressure buildup in the reservoir, meaning that the existence of intraformational barriers suppresses the vertical communication of the pressure in the reservoir. This can be better understood from Fig. 6c, showing that the maximum pressure buildup was observed at locations far from the top of the reservoir. Furthermore, we observed a sharp attenuation of $\Delta P_{top}$ after injection stops (see Fig. 6b). Figure 7 illustrates the variation of pressure along the reservoir depth at the end of injection and post-injection for one of the scenarios. It can be noted that the pressure plume is mainly accumulated at the greater reservoir depths, while the pressure of the upper layers remains almost unchanged during CO$_2$ storage.

### Sensitivity analysis

We also examined the level of the sensitivity of CO$_2$ plume shape and pressure response of the confining zone to uncertain parameters including *NTG*, $cl_x$, $K_{v}/K_{h}$, $P_{ce,sand}$, and $P_{ce,shale}$. A visual display of the sensitivity analysis is depicted in the tornado chart in Fig. 8. This chart represents the contribution of uncertain parameters to the response variables including $S_{CO_2}$, $L_{y}/H$, $L_{x}/L$, *CM*/*H*, $\Delta P_{max}$, and $\Delta P_{top}$. To build the tornado chart, one parameter at a time was varied between its lower and upper values taken from their uniform distribution, while other variables were kept at their median values. Then, the model response were laid out in the tornado chart. The degree of sensitivity of the uncertainty metrics to the input variables can be quantified by the bar lengths corresponding to each variable. As can be seen, the permeability anisotropy ($K_{v}/K_{h}$) of sand has no effects on the vertical migration of CO$_2$ plume ($L_{y}/H$ and *CM*/*H*), which is yet highly controlled by the correlation length ($cl_x$) and density (*NTG*) of capillary barriers. Although buoyancy-driven migration of CO$_2$ in homogeneous reservoir models has been shown to be highly influenced by the permeability anisotropy in previous studies^39^, our results delineate that the lateral extension and density of capillary barriers have more influential impact on retarding the upward migration of CO$_2$. According to the tornado chart, it was observed that $P_{ce,sand}$ has minimal impacts on the configuration of CO$_2$ plume (i.e., $L_{y}/H$, $L_{x}/L$, and *CM*/*H*) and pressure buidlup (i.e., $\Delta P_{max}$, and $\Delta P_{top}$). This finding suggests that the capillary entry pressure of sand bodies does not play a key role in controlling the migration of CO$_2$ plume and pressure propagation in a heterogeneous reservoir with capillary barriers. However, it is an influential parameter controlling CO$_2$ plume geometry in homogeneous reservoirs^40^. It was observed that lower values of $P_{ce,shale}$ promote the upward migration of CO$_2$ plume (i.e., $L_{y}/H$), leading to reduced lateral migration (i.e., $L_{x}/L$). In general, according to the sensitivity analysis, it is evident that the CO$_2$ plume geometry and pressure propagation are highly influenced by *NTG* and $cl_x$ in a heterogeneous medium.

The box plots shown in Fig. 9 represent the dependence of CO$_2$ saturation ($S_{CO_2}$) and aspect ratio of the plume ($AR_{plume}$, which is $\frac{L_{x}}{L_{y}}$), on 1-*NTG* and $cl_x/L$. According to Fig. 9a, we observed a positive correlation between the median value of $S_{CO_2}$ with the length of barriers and their volume fraction in the confining zone. However, $S_{CO_2}$ dependence on *NTG* appears to be more pronounced than that on $cl_x/L$. According to Fig. 9c and d, $AR_{plume}$ median values show a positive correlation with *NTG* and $cl_x/L$, while the correlation with *NTG* is more significant. The findings indicate that there are no substantial changes in the aspect ratio and spatial distribution of the plume when the lateral extent of the barriers exceeds 40$\%$ of the formation’s length.

**Figure 10:**
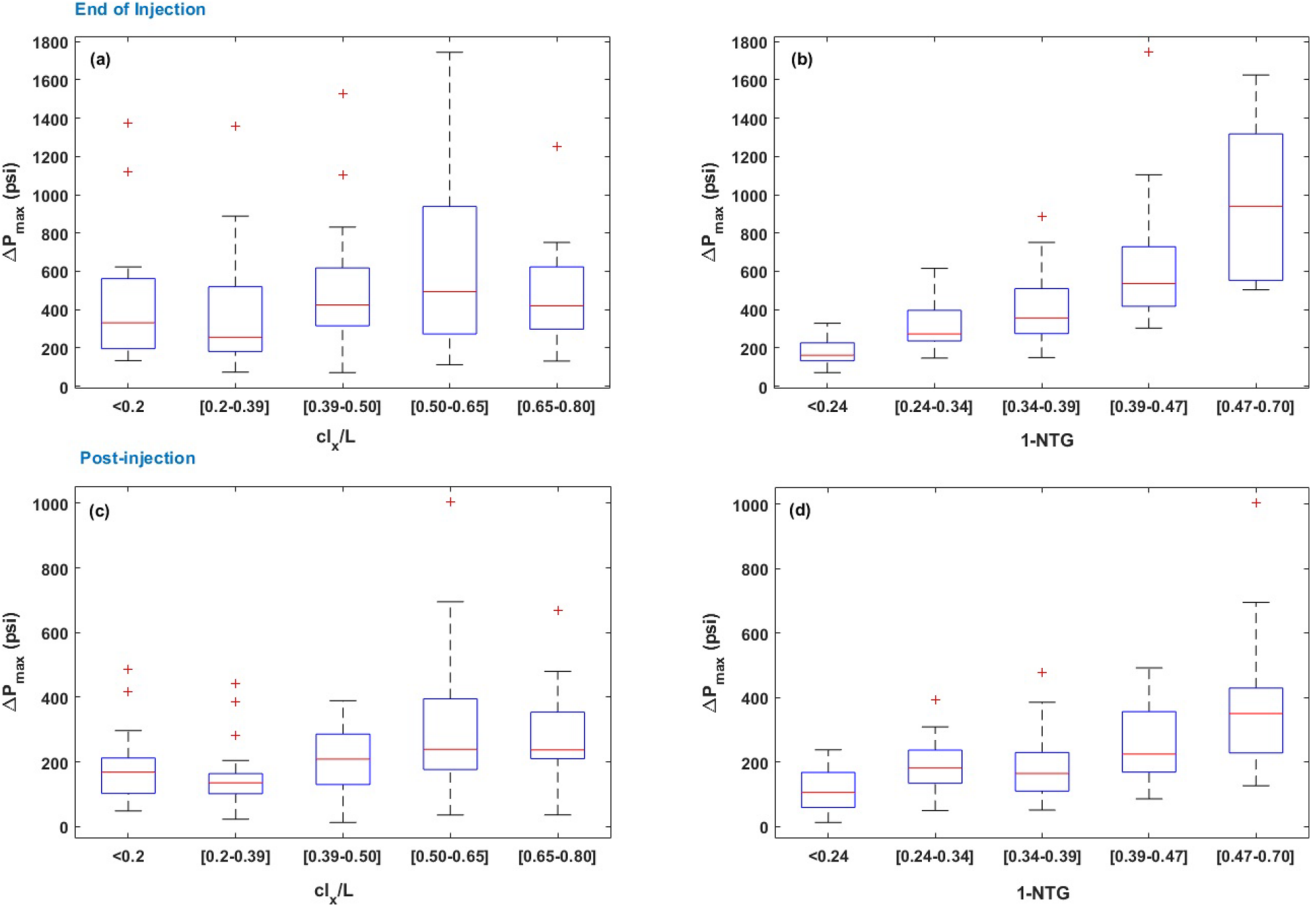
Box plots representing the dependence of $\Delta P_{max}$ on *NTG* and $cl_x/L$ at the end of injection and 200 years post-injection.

**Figure 11:**
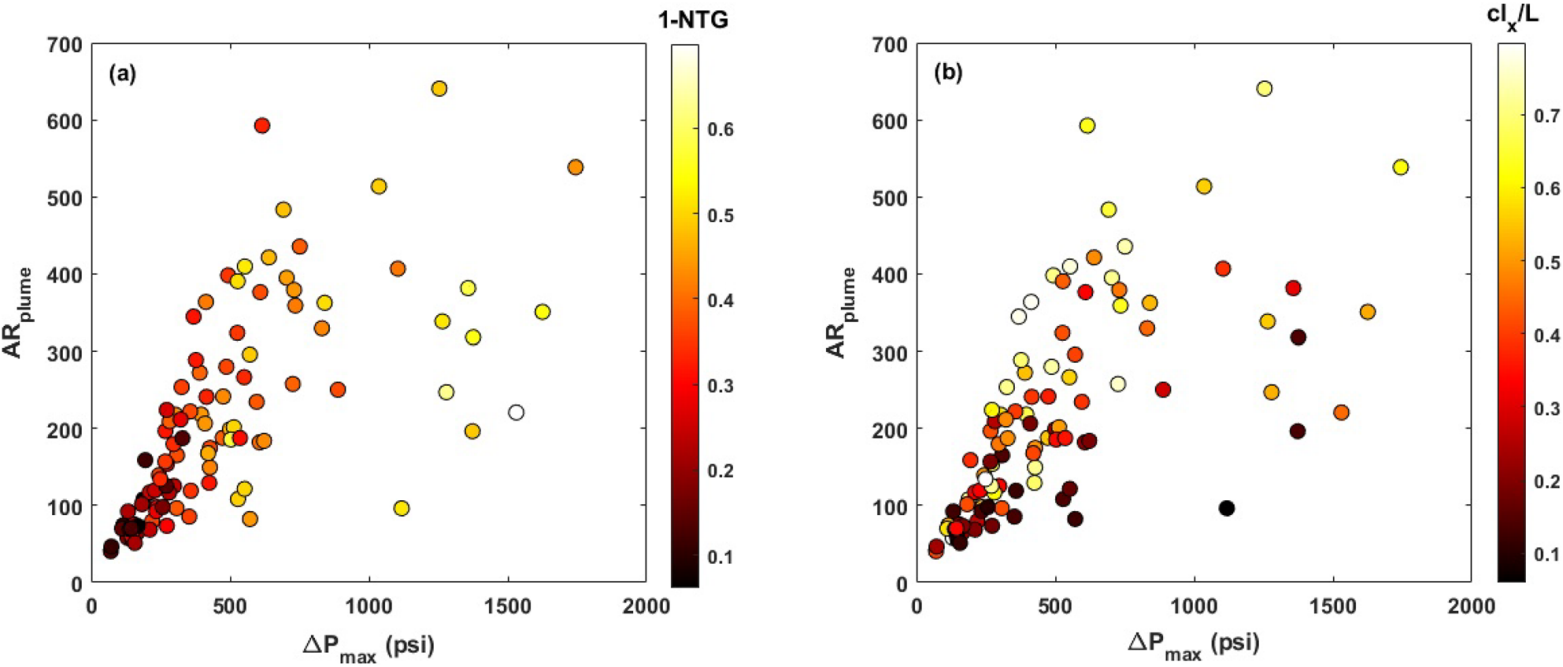
Variations in the aspect ratio of the plume ($AR_{plume}$) as a function of maximum pressure buildup ($\Delta P_{max}$) as well as *NTG* and $cl_x/L$.

The box plots shown in Fig. 10 represent the dependence of $\Delta P_{max}$ on 1-*NTG* and $cl_x/L$ at the end of injection and 200 years post-injection. We observed an increasing trend in the maximum pressure buildup as the barrier length and their volume fraction increase, while the correlation with *NTG* is more pronounced. Considering the sensitivity of the formation’s response to *NTG*, we recommend that the characterization of a composite confining system of heterogeneous barriers should be heavily focused on the quantification of this characteristic.

Figure 11 represents the variations in the plume aspect ratio ($AR_{plume}$) as a function of maximum pressure buildup $\Delta P_{max}$ as well as the role of *NTG* and $cl_x/L$ on their correlation. According to these results, high aspect ratios correspond to high pressure buildups in the formation and they appear at large barrier lengths and large volume fractions of barriers. Although large barriers or a high density of barriers can promote the lateral extension of the plume, they can also lead to a higher pressure buildup, as pressure communication in the vertical direction is limited in the presence of barriers. However, according to Fig. 6, it should be noted that pressure accumulates mainly in the deeper parts of the confining zone.

## Summary

This study investigates the containment of CO$_2$ in regards to its vertical migration within lithologically heterogeneous reservoir rocks composed of stacked fine-grained barriers interbedded within sand units known as composite confining systems^5^. The reservoir-scale two-phase flow simulations presented here are representative of the pressure-driven and buoyancy-driven flow of CO$_2$ occurring during injection and post-injection in saline aquifers. Heterogeneous facies models were generated through geostatistically populating horizontal discontinuous shale barriers into sand bodies. Numerous geologic models were generated encompassing various combinations of uncertain parameters, including *NTG*, $cl_x$, $K_{v}/K_{h}$, $P_{ce,sand}$, and $P_{ce,shale}$. We studied the impact of heterogeneous composite systems on the pressure- and buoyancy-driven migration of CO$_2$, the effectiveness of confinement, and the formation’s pressure response.

According to the simulation results, we observed that the existence of barrier layers exerts a significant influence on the vertical migration of the plume and the pressure propagation in the composite system. The dispersed flow path of CO$_2$ plume, controlled by the spatial heterogeneity of capillary barriers, is more pronounced after the cessation of injection when the plume migrates upward due to the effect of buoyancy forces. Based on the parametric sensitivity analysis, it has been demonstrated that the lateral continuity of discontinuous barriers (i.e., $cl_x$) and their volume fraction (i.e., *NTG*) play significant roles in determining the shape of the CO$_2$ plume and vertical pressure communication in the system. With increasing the fraction of barriers and their horizontal length, the vertical movement of CO$_2$ plume becomes restricted while its lateral movement is encouraged. Furthermore, the plume shape and pressure response and overall retention capacity of a composite system were found to be more susceptible to changes in *NTG* than $cl_x$. Hence, *NTG* should be prioritized as the key factor for geologic characterization of composite systems, given its significant influence on the dynamic response of such systems to CO$_2$ injection.

With regard to the findings of this study, it is reasonable to suggest that formations containing capillary barriers are effective in containing the injected CO$_2$ plume within the formation, as heterogeneity serves to limit the reliance of the formation seal as the only mechanism for containment. Even though the composite system considered in this study acts as the injection zone, the efficacy of such a system for CO$_2$ containment at the post-injection stage, where plume buoyant migration is dominant, has been demonstrated. A study by Bump et al.^5^ has provided further demonstration of the effectiveness of composite systems as a confining zone. The 3D reservoir-scale flow simulation in their study has shown that the migration of CO$_2$ plume could be predominantly horizontal with minimal intrusion into the confining zone with the extent of the capillary barriers occupying at least 6$\%$ of the reservoir domain.

## Data Availability

The datasets used and/or analysed for this study are available from the corresponding author upon reasonable request.

## References

[1] Fan M (2014). Reservoir stratigraphic heterogeneity within the Lower Cretaceous Muddy Sandstone in the Powder River Basin, northeast Wyoming, U.S.A.: Implications for carbon dioxide sequestration. Rocky Mt. Geol..

[2] Hovadik JM, Larue DK (2010). Stratigraphic and structural connectivity. Geol. Soc. Lond. Special Publ..

[3] Hurst A (1992). Sedimentary Flow Units in Hydrocarbon Reservoirs: Some Shortcomings and a Case for High-Resolution Permeability Data.

[4] Begg, S. & King, P. Modelling the effects of shales on reservoir performance: Calculation of effective vertical permeability. In *SPE Reservoir Simulation Conference All Days*10.2118/13529-MS (1985).

[5] Bump AP (2023). Composite confining systems: Rethinking geologic seals for permanent CO$$_2$$ sequestration. Int. J. Greenhouse Gas Control.

[6] Hovorka SD, Doughty C, Benson SM, Pruess K, Knox PR (2004). The impact of geological heterogeneity on CO$$_2$$ storage in brine formations: A case study from the Texas Gulf Coast. Geol. Soc. Lond. Special Publ..

[7] Flett, M., Gurton, R. & Taggart, I. Heterogeneous saline formations: Long-term benefits for geo-sequestration of greenhouse gases. In *Greenhouse Gas Control Technologies*, vol. 7, 501–509. 10.1016/B978-008044704-9/50051-3 (Elsevier Science Ltd, 2005).

[8] Gibson-Poole, C. M. *et al.* Understanding stratigraphic heterogeneity: A methodology to maximize the efficiency of the geological storage of CO$$_2$$. In *Carbon Dioxide Sequestration in Geological Media-State of the Science*10.1306/13171248St593385 (American Association of Petroleum Geologists, 2009).

[9] Akai T, Kuriyama T, Kato S, Okabe H (2021). Numerical modelling of long-term CO$$_2$$ storage mechanisms in saline aquifers using the Sleipner benchmark dataset. Int. J. Greenhouse Gas Control.

[10] Cossins T, Mishra A, Haese RR (2023). The feasibility of enhanced pore space utilization in CO$$_2$$ storage reservoirs using an artificially emplaced Si-gel flow barrier. Sci. Rep..

[11] Ni H, Bakhshian S, Meckel TA (2023). Effects of grain size and small-scale bedform architecture on CO$$_2$$ saturation from buoyancy-driven flow. Sci. Rep..

[12] Ajayi T, Gomes JS, Bera A (2019). A review of CO$$_2$$ storage in geological formations emphasizing modeling, monitoring and capacity estimation approaches. Petrol. Sci..

[13] Song J, Zhang D (2013). Comprehensive review of caprock-sealing mechanisms for geologic carbon sequestration. Environ. Sci. Technol..

[14] Skerlec, G. M. Evaluating top and fault seal. In *Exploring for Oil and Gas Traps*10.1306/TrHbk624C11 (American Association of Petroleum Geologists, 1999).

[15] Woods AW, Farcas A (2009). Capillary entry pressure and the leakage of gravity currents through a sloping layered permeable rock. J. Fluid Mech..

[16] Flett M, Gurton R, Weir G (2007). Heterogeneous saline formations for carbon dioxide disposal: Impact of varying heterogeneity on containment and trapping. J. Petrol. Sci. Eng..

[17] Saadatpoor E, Bryant SL, Sepehrnoori K (2010). New trapping mechanism in carbon sequestration. Transp. Porous Media.

[18] Hesse MA, Woods AW (2010). Buoyant dispersal of CO$$_2$$ during geological storage. Geophys. Res. Lett..

[19] Trevisan L (2017). Imaging and quantification of spreading and trapping of carbon dioxide in saline aquifers using meter-scale laboratory experiments. Water Resour. Res..

[20] Krevor SCM, Pini R, Li B, Benson SM (2011). Capillary heterogeneity trapping of CO$$_2$$ in a sandstone rock at reservoir conditions. Geophys. Res. Lett..

[21] Mishra A, Haese RR (2020). Quantification of the turning point saturation for cross bedded CO$$_2$$ storage reservoirs. Int. J. Greenhouse Gas Control.

[22] Gershenzon NI, Ritzi RW, Dominic DF, Mehnert E, Okwen RT (2017). Capillary trapping of CO$$_2$$ in heterogeneous reservoirs during the injection period. Int. J. Greenhouse Gas Control.

[23] Zhao B, MacMinn CW, Huppert HE, Juanes R (2014). Capillary pinning and blunting of immiscible gravity currents in porous media. Water Resour. Res..

[24] Perrin J-C, Benson S (2010). An experimental study on the influence of sub-core scale heterogeneities on CO$$_2$$ distribution in reservoir rocks. Transp. Porous Media.

[25] MacDonald AC, Halland EK (1993). Sedimentology and shale modeling of a sandstone-rich fluvial reservoir: Upper Statfjord Formation, Statfjord Field, Northern North Sea. AAPG Bull..

[26] Al-Mudhafar WJ (2019). Bayesian kriging for reproducing reservoir heterogeneity in a tidal depositional environment of a sandstone formation. J. Appl. Geophys..

[27] Bakhshian S, Hosseini SA (2019). Pore-scale analysis of supercritical CO$$_2$$-brine immiscible displacement under fractional-wettability conditions. Adv. Water Resour..

[28] Holtz, M. Residual gas saturation to aquifer influx: A calculation method for 3-D computer reservoir model construction. In *SPE Unconventional Resources Conference/Gas Technology Symposium All Days*10.2118/75502-MS (2002).

[29] CMG-GEM. Advanced compositional and unconventional reservoir simulator. (Computer Modeling Group Ltd, 2012).

[30] Bakhshian S, Shariat A, Raza A (2022). Assessment of CO$$_2$$ storage potential in reservoirs with residual gas using deep learning. Interpretation.

[31] Bakhshian S, Hosseini SA, Lake LW (2020). CO$$_2$$-brine relative permeability and capillary pressure of tuscaloosa sandstone: Effect of anisotropy. Adv. Water Resour..

[32] Li B, Tchelepi HA, Benson SM (2013). Influence of capillary-pressure models on CO$$_2$$ solubility trapping. Adv. Water Resour..

[33] Land C (1968). Calculation of imbibition relative permeability for two- and three-phase flow from rock properties. SPE J..

[34] Pollyea RM (2016). Influence of relative permeability on injection pressure and plume configuration during CO$$_2$$ injections in a mafic reservoir. Int. J. Greenhouse Gas Control.

[35] Wu H, Lubbers N, Viswanathan HS, Pollyea RM (2021). A multi-dimensional parametric study of variability in multi-phase flow dynamics during geologic CO$$_2$$ sequestration accelerated with machine learning. Appl. Energy.

[36] Elnaggar OM, Temraz MG (2018). Miocene reservoir rocks: Pore throat size distribution as a strong controller on petrophysical attributes is a reflection of facies change. J. Petrol. Explor. Prod. Technol..

[37] Leverett M (1941). Capillary behavior in porous solids. Trans. AIME.

[38] Freyberg DL (1986). A natural gradient experiment on solute transport in a sand aquifer: 2. Spatial moments and the advection and dispersion of nonreactive tracers. Water Resour. Res..

[39] Yu X (2020). Impact of reservoir permeability, permeability anisotropy and designed injection rate on CO$$_2$$ gas behavior in the shallow saline aquifer at the cami field research station, brooks, alberta. Nat. Resour. Res..

[40] Wu H, Jayne RS, Pollyea RM (2018). A parametric analysis of capillary pressure effects during geologic carbon sequestration in a sandstone reservoir. Greenhouse Gases Sci. Technol..

